# Breaching the Blood–Brain Tumor Barrier for Tumor Therapy

**DOI:** 10.3390/cancers13102391

**Published:** 2021-05-15

**Authors:** Fabrizio Marcucci, Angelo Corti, Andrés J. M. Ferreri

**Affiliations:** 1 Department of Pharmacological and Biomolecular Sciences, University of Milan, 20132 Milan, Italy; 2 Division of Experimental Oncology, Tumor Biology and Vascular Targeting Unit, IRCCS San Raffaele Scientific Institute, 20132 Milan, Italy; 3 Faculty of Medicine and Surgery, Università Vita-Salute San Raffaele, 20132 Milan, Italy; 4 Lymphoma Unit, Department of Onco-Hematology, IRCCS San Raffaele Scientific Institute, 20132 Milan, Italy; ferreri.andres@hsr.it

**Keywords:** brain tumors, blood–brain barrier, blood–brain barrier permeabilization, drug delivery, cancer therapy, clinical trials

## Abstract

**Simple Summary:**

The blood–brain tumor barrier (BBTB) represents a major obstacle for the delivery of anticancer drugs to tumors of the central nervous system. Various approaches have been so far developed for overcoming this obstacle and for increasing anti-cancer drug concentrations in tumor tissues. This review is focused on the latest clinical advances and achievements in breaching the BBTB for primary and secondary brain tumor therapy.

**Abstract:**

Tumors affecting the central nervous system (CNS), either primary or secondary, are highly prevalent and represent an unmet medical need. Prognosis of these tumors remains poor, mostly due to the low intrinsic chemo/radio-sensitivity of tumor cells, a meagerly known role of the microenvironment and the poor CNS bioavailability of most used anti-cancer agents. The BBTB is the main obstacle for anticancer drugs to achieve therapeutic concentrations in the tumor tissues. During the last decades, many efforts have been devoted to the identification of modalities allowing to increase drug delivery into brain tumors. Until recently, success has been modest, as few of these approaches reached clinical testing and even less gained regulatory approval. In recent years, the scenario has changed, as various conjugates and drug delivery technologies have advanced into clinical testing, with encouraging results and without being burdened by a heavy adverse event profile. In this article, we review the different approaches aimed at increasing drug delivery to brain tumors, with particular attention to new, promising approaches that increase the permeability of the BBTB or exploit physiological transport mechanisms.

## 1. Introduction

Despite some recent progress, the overall prognosis of tumors that primarily arise in or metastasize to the central nervous system (CNS) remains largely unsatisfactory. The poor CNS bioavailability of most anti-cancer agents plays a crucial role in this regard. This is due to the presence of the blood–brain barrier (BBB), which protects the CNS from potentially harmful blood-borne substances, including anticancer drugs, and regulates influx and efflux of molecules. Patients with gliomas, or other neoplasms of the CNS, such as brain metastases or lymphomas, would greatly profit from enhanced access of drugs into the CNS. Thus, overcoming this barrier in brain tumors (referred to as the blood–brain tumor barrier, BBTB) with innovative strategies has become an important therapeutic goal. Literature on the biological and molecular characteristics of the BBTB is rapidly growing, and progress in gaining knowledge on the physiology and pathophysiology of this barrier has provided the background for the development of new approaches that have been addressed in a number of clinical studies. This article is not focused on the biological and molecular details of the BBTB, which can be found in excellent recent reviews [1,2,3,4]. Instead, we review the available literature on novel treatments for primary or secondary brain cancers that overcome the BBTB and discuss in details some recent approaches that hold considerable promise. The molecular bases, targets, and drug delivery systems, as well as the design and results of clinical trials, are critically analyzed, and future perspectives are discussed.

## 2. The Blood–Brain Barrier (BBB) and the Blood–Brain Tumor Barrier (BBTB)—Some Fundamentals

Different cell types contribute to the BBB: endothelial cells, pericytes, astrocytes, microglial cells and neurons, which, together, constitute the neurovascular unit (NVU) [3]. Endothelial cells are the main component of the BBB and display some unique properties compared to endothelial cells in other compartments: they have continuous intercellular tight junctions (TJ), lack fenestrations and undergo very low rates of transcytosis, thereby minimizing any kind of paracellular and transcellular transport through the endothelial cell layer [1]. Moreover, low expression of leukocyte adhesion molecules keeps at a minimum immune cell infiltration into the brain. Likewise, astrocytes, pericytes, microglia cells and neurons exhibit important specializations, and recent investigations on single-cell components of the NVU have confirmed the organotypic molecular signatures of these cells [5,6]. In addition, the basement membrane, produced by the different cell types of the NVU through the secretion of extracellular matrix (ECM) molecules, contributes to the BBB, by keeping the different cell types in place and regulating their intercellular crosstalk [3]. The BBB is so efficient in sealing off the CNS that only small, lipophilic molecules, with an Mr < 400 Da, can enter the CNS through passive diffusion across the lipid bilayer membranes of endothelial cells [7]. Otherwise, exchange of molecules between the blood and brain parenchyma is the task of specific transporters that regulate the translocation of selected molecules across the endothelium [8,9].

The BBB is not an immutable entity, experiencing significant changes in response to pathologies affecting the CNS. In the presence of a primary or secondary brain tumor, for example, it is now customary to refer to a BBTB rather than to a BBB, to evidence the differences between the two [4]. Some of these differences are the consequence of the abnormal angiogenesis that characterizes tumor formation, with neoangiogenic tumor vessels expressing markers that are not present in quiescent endothelial cells [10]. Brain tumorigenesis is also accompanied, similarly to other tumors arising outside the CNS, by inflammatory changes that are closely intertwined with neoangiogenic changes [11]. Moreover, existing vessels may be compressed by the growing tumor, thereby impairing blood flow [12], or they may be co-opted by the tumor [13]. Altogether, these and other alterations lead to increased leakiness of the BBTB, which, however, is very heterogeneous even within individual malignant foci [14] and, therefore, inappropriate to be exploited for homogeneous drug delivery within affected brain areas. Furthermore, tumor cells can be detected by histopathologic analysis not only in areas characterized by high permeability and detected by neuroimaging techniques, but also far from these areas. In the present article, which has its main focus on the therapy of brain tumors, we will always utilize the term BBTB unless we refer to studies regarding non-neoplastic diseases. In these cases, the term BBB will be used.

Overall, the BBB/BBTB represents an especially difficult barrier to overcome within the more general context of delivering drugs out of the circulation into diseased tissues, whether affected by cancer or other pathologies [15,16].

## 3. Approaches to Overcome the BBTB for Brain Tumor Therapy

**Figure 1:**
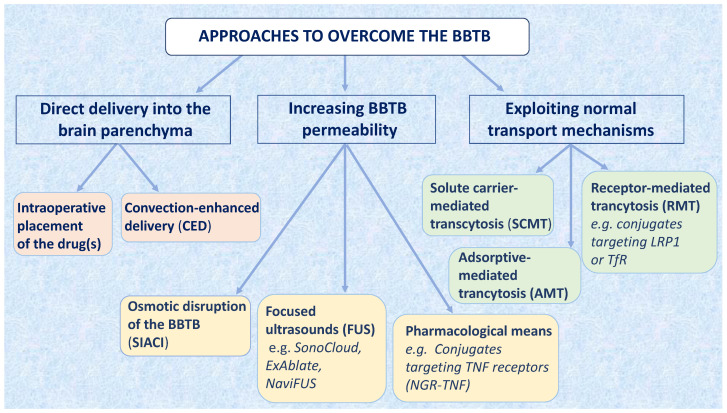
Approaches to overcome the BBTB. Three main classes of approaches to overcome the BBTB are shown. Each class is divided in different subclasses. Abbreviations: BBTB, blood–brain tumor barrier; FUS, focused ultrasound; SCMT, solute carrier-mediated transcytosis; SIACI, superselective intraarterial cerebral infusion; Tfr, transferrin receptor; TNFR, tumor necrosis factor receptor.

The BBTB allows to deliver to tumor tissues only a few highly lipophilic compounds of low Mr (e.g., thiotepa and temozolomide). Other drugs are delivered at high doses, allowing sufficient tumor penetration by passive diffusion following a concentration gradient between intra- and extra-vascular spaces, but at the expense of higher side effects [17,18]. The development of strategies to overcome the BBTB could expand the spectrum of usable drugs, with a potential improvement in therapeutic efficacy and tolerability (Figure 1). The implementation of these strategies is expected to improve the delivery of drugs both against primary brain tumors as well as tumors that metastasize to the brain [19,20,21].

### 3.1. Delivering Drugs Directly into the Brain

#### 3.1.1. Wafers Impregnated with Anticancer Drugs

A first approach rests on delivering the desired compound(s) directly into the tumor parenchyma. This can be achieved through placement of a drug in the resection cavity created during a surgical intervention of tumor debulking. For this purpose, polymer wafers impregnated with a chemotherapeutic drug are used and placed in the cavity [22]. Wafers embedded with carmustine (e.g., Gliadel^®^ wafers), which allow a sustained drug release for at least 5 days while the wafer is degraded in 2–3 weeks, have received regulatory approval for the treatment of glioblastoma by the US Food and Drug Administration (FDA) and other regulatory authorities in 2003. In general, efficacy studies have demonstrated modest survival advantages [23,24], but a controlled, propensity-matched analysis has shown a significant prolongation of progression-free survival (PFS) in a multicenter cohort of patients with newly diagnosed glioblastoma treated with Gliadel^®^ wafer implantation followed by standard chemoradiotherapy [25]. An ongoing randomized phase III trial (JCOG1703) is addressing the contribution of Gliadel^®^ to maximal tumor resection followed by chemoradiotherapy in patients with glioblastoma [26]. Clinical trials with Gliadel^®^, retrievable from the clinicaltrials.gov website, are reported in Table 1. Research in this setting is focused on the use of new impregnated drugs and the development of novel materials and device technologies [27].

#### 3.1.2. Convection-Enhanced Delivery (CED)

CED represents a valid alternative to infuse a compound directly in the tumor parenchyma. Here, a syringe pump and a cannula implanted into the tumor creates a pressure gradient, thereby allowing distribution of the compound throughout contiguous brain areas [28]. Several clinical studies have demonstrated the safety and feasibility of a variety of agents injected by CED (e.g., recombinant fusion proteins, oncolytic viruses, monoclonal antibodies (mAbs) and liposomal drugs); however, the large variability in the techniques, infusates and hardware used make it difficult to draw reliable conclusions on antitumor efficacy. Despite these limitations, encouraging results have been reported in some early-phase clinical studies. Diffuse intrinsic pontine glioma, a universally fatal tumor of pediatric patients, is a suitable candidate for CED-based treatment because it is constrained within a limited anatomical compartment, without the tissue inhomogeneities typically observed in other brain tumors managed with cytoreductive resection. The CED of ^124^I-labeled anti-B7-H3 mAb 8H9 has been performed in children with diffuse intrinsic glioma in a dose-escalation phase I trial, showing a good safety profile and high intratumoral dosing with negligible systemic exposure [29]. A recently reported phase I trial has demonstrated that nimustine delivered by CED in combination with temozolomide was safe and active in patients with recurrent diffuse intrinsic pontine glioma (*n* = 11) and other brainstem gliomas (*n* = 5) [30]. Most treated patients experienced worsening of transient symptoms due to local edema; tumor size reduction was recorded in most patients who received the highest assessed dose.

The CED of recombinant proteins consisting of a bacterial toxin, such as the *Pseudomonas aeruginosa* exotoxin, fused to a cytokine (such as transforming growth factor (TGF), interleukin (IL)-4 or IL-13) has been investigated in patients with recurrent high-grade gliomas [31,32,33]. Phase I trials have demonstrated that the CED of these compounds is associated with grade 3–4 side effects in one-third of patients, with no deaths due to toxicity [31,32]. In the PRECISE randomized phase III trial [34], the IL-13-*Pseudomonas* exotoxin A conjugate (termed IL13-PE38QQR) has produced results similar to those of the Gliadel^®^ wafer, in 296 patients with recurrent glioblastoma, as a result of which the clinical development of this compound was discontinued. The CED of recombinant, nonpathogenic polio-rhinovirus chimera (PVSRIPO), a virus that recognizes CD155 in tumors cells and the microenvironment, has been safe in 61 patients with recurrent glioblastoma [35], with a 3-year OS of 21%, which is better than the survival rates reported in historical controls; these results deserve confirmation in more advanced trials.

The CED of chemotherapeutic drugs is an effective way to overcome the BBTB, which could result in higher antitumor activity as well as in higher toxicity. The CED of paclitaxel is a good example of this balance [36,37]. Paclitaxel is an anticancer drug inefficacious in patients with recurrent glioblastoma, as it crosses the BBTB to a minimal degree. When administered by CED, this taxane has been associated with an overall response rate (ORR) of 73% in these patients but, at the same time, with important complications, such as chemical meningitis, infectious complications, transient neurological impairment, and skin necrosis. Thus, optimization of this approach to reduce related toxicity should be explored further.

Therapeutic failures with CED, mostly those reported in the PRECISE trial [34], have been attributed to ineffective drug infusion resulting from suboptimal catheter positioning [38]. In fact, all criteria of the catheter positioning guidelines were met only in half of the treated patients, which represents an important limitation in a multicenter setting. Despite these limitations, CED remains the most intensively investigated approach to bypass the BBTB, with several ongoing clinical trials (Table 1).

It should be noted, however, that this method, as the other methods herein described based on local drug delivery to a specific brain area, will deliver the chemotherapy only to the tumor bulk and not to distant, migrating tumor cells (which may still reside behind the BBTB).

### 3.2. Increasing the Permeability of the BBTB

Some strategies have been designed to transiently disrupt the BBTB in order to increase its permeability, allowing the extravasation of drugs into the brain tumor parenchyma.

#### 3.2.1. Osmotic Disruption of the BBTB

This approach rests on the temporary disruption of the BBTB by carotid or vertebral artery injection of a hyperosmotic solution, generally a highly concentrated solution of mannitol, just before drug administration by the same route [39]. The administration of the hyperosmotic solution causes water to be withdrawn from the endothelial cells lining the BBTB, with the consequent shrinkage and opening of the inter-endothelial junctions, which, in turn, leads to increased transport of systemically administered drugs across the BBTB. Osmotic disruption of the BBTB is transient and reversible [40]. This approach is referred to as superselective intraarterial cerebral infusion (SIACI). The locoregional administration protocol is required in order to avoid systemic effects. However, given the invasiveness of the procedure, it comes to no surprise that it requires hospitalization, patient sedation and can be accompanied by adverse events (AEs), such as neurological deficits, strokes, seizures and new tumor-nodule formation [41], mostly due to a transient edema that ensues the increased bulk fluid influx.

SIACI has been used to deliver mAbs. SIACI of cetuximab, an anti-epidermal growth factor receptor (EGFR) mAb, has been safe and well tolerated in patients with recurrent glioblastoma in a phase I/II trial, with no dose-limiting toxicity (DLT) up to 250 mg/m^2^ [42]; a phase II trial is ongoing (Table 2). When administered by SIACI, bevacizumab, an anti-vascular endothelial growth factor (VEGF) mAb, has been associated with a good safety profile and some cases of tumor regression in patients with recurrent gliomas [43,44].

Reversible BBTB disruption by intra-arterial infusion of mannitol followed by intra-arterial methotrexate has been investigated in patients with newly diagnosed or relapsed primary CNS lymphoma, in a few institutions with adequate expertise. This strategy has been associated with a 58% complete response (CR) rate, 5-year PFS of 31% and acceptable morbidity and neurotoxicity [45]. Overall, BBTB disruption shows an efficacy similar to that of standard high-dose-methotrexate-based treatments, but it is a procedurally intensive treatment, requiring monthly intravascular interventions under general anesthesia over the course of 1 year. Osmotic disruption by SIACI is currently being investigated in several clinical trials in combination with various drugs in patients with primary brain tumors or brain metastases (Table 2).

#### 3.2.2. Ultrasound/Focused Ultrasound

**Figure 2:**
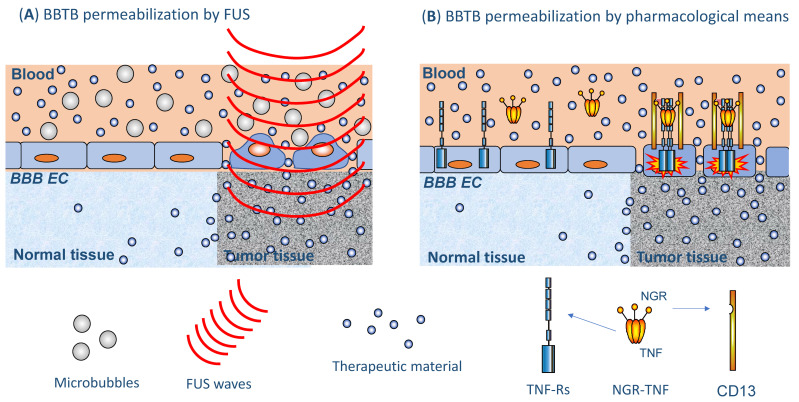
Drug delivery to the brain tumors through permeabilization of the BBTB. This figure shows two different approaches to enhance drug delivery through the BBTB. On the left side (**A**), permeabilization is achieved by means of focused ultrasound (FUS) in combination with microbubbles. It is the ultrasound (US) focusing that endows this approach with selectivity for the BBTB at desired sites of the brain. On the right side (**B**), permeabilization is achieved by means of a pharmacologically active compound that exerts its effect through disruption of TJs (e.g., NGR-TNF, a peptide-TNF fusion product that targets CD13-positive tumor vasculature), optimally only on endothelial cells of the BBTB.

Low-intensity pulsed ultrasound (US) can be directed to discrete areas of the brain by different means and is, therefore, referred to as focused US (FUS) [7,46]. This technique allows targeting and destroying desired cell clusters by thermocoagulation while sparing adjacent cells and tissues. FUS is combined with intravenous injection of microbubbles (1–10 μm diameter), which consist of lipid or albumin shells that encapsulate gaseous material [7] (Figure 2A). In the selected area of the brain, the microbubbles vibrate in response to the US waves, leading to the transient (4–6 h) disruption and increased permeability of the BBTB [47]. There are different classes of commercially available microbubbles but none appears to be clearly superior to another [48]. Furthermore, there are different FUS devices for BBTB breaching that are being investigated in clinical trials. A first approach foresees the implantation of a transducing device in the skull (SonoCloud^®^, CarThera) in order to minimize a major problem of US technology in BBTB opening; i.e., the thickness of the skull bone in humans, which distorts and absorbs much US energy. SonoCloud is, in fact, an unfocused, low-intensity, pulsed US system that allows for diffuse, transient opening of the BBTB. It is actually the site of implantation of the device and the sonicated area that allow it be considered as a focused approach. The device can be activated repeatedly allowing the repetitive administration of drugs into the desired area of the brain. It has been shown to be safe and afford efficient, transient opening of the BBB in nonhuman primates over a 3-month period without behavioral, immunological or neurological consequences [49,50]. SonoCloud followed by intravenous carboplatin injection has been tested in 21 patients with recurrent glioblastoma enrolled in a phase I/II trial; the BBTB has been disrupted monthly with pulsed US in combination with microbubbles, with transient and manageable AEs [51]. No carboplatin-related neurotoxicity was observed. Patients with no or poor BBTB disruption visible on magnetic resonance imaging (MRI) had a median PFS of 3 months and a median overall survival (OS) of 9 months. Interestingly, patients with clear BBTB disruption had a median PFS of 4 months and a median OS of 13 months [52]. These latter results compare favorably with historical data. In order to improve these outcomes, the authors intend to develop a device with a larger sonication area allowing to extend drug delivery to the largest possible tumor region. SonoCloud is being investigated in several other clinical trials for BBTB opening (Table 2). The results obtained with this device appear promising, but on the downside is the need to implant it in the skull of the patients—an apparently well-tolerated but nevertheless invasive procedure.

A second approach foresees the use of an extracorporeal fixed MRI-guided US-emitting device (ExAblate^®^, InSightec). The device is embedded within a diagnostic MRI scanner, which guides and monitors the USs to the desired area of the brain [53,54]. ExAblate^®^ has been investigated successfully for several clinical indications, beyond BBTB disruption. It has been successfully tested in several animal models and the results of two clinical studies published on this approach, performed in patients with non-neoplastic CNS diseases, have shown that the BBB was safely, reversibly and repeatedly opened in all patients [55,56]. No AEs have been detected post-sonication and patients have tolerated up to 17 FUS treatments. BBB opening with an average of 95% of the targeted FUS volume has been achieved and this has been followed by BBB closure within 24 h. The safety and feasibility of this procedure in patients with brain tumors are currently addressed in several clinical trials (Table 2), whereas efficacy studies are not yet ongoing.

Interestingly, some clinical studies, not reported in Table 2 because they are beyond of the scope of the article, describe the use of FUS to induce direct antitumor effects on brain tumors independently of its effects on the BBTB. This is not surprising since ExAblate^®^ is being investigated for direct antitumor effects on many extracranial tumors and has received FDA approval for the treatment of uterine fibroids [57]. This suggests the possibility of using FUS both for BBTB opening as well as for inducing direct antitumor effects, most likely by tailoring key parameters such as frequency and acoustic pressure for each of the two objectives, which could be pursued contemporarily.

A third FUS-based approach uses a frameless neuronavigation-guided device (NaviFUS^®^), which generates FUS treating units for covering the desired brain volume (e.g., tumor volume). The location of the treating units is determined using software from CT/MRI images of the patient that are collected before treatment. Guidance of FUS energy to the selected treatment area is achieved by means of a high-precision neuro-navigation tracking system [58]. The NaviFUS^®^ system has been investigated in one clinical trial for BBTB opening [59].

Overall, US-based approaches seem of considerable promise. They are less invasive than CED and osmotic BBTB disruption, exhibit the possibility of precisely targeting discrete regions of the brain and appear fully reversible [49,52,60,61,62,63]. While the safety of these approaches looks very promising, more studies are required to evaluate the risks associated with repeated treatments, with or without delivery of a therapeutic agent [63]. Based on these encouraging features, results of ongoing clinical studies (Table 2) are expected to promote this strategy to more advanced levels.

#### 3.2.3. Increasing BBTB Permeability by Pharmacological Means

Historically, this is one of the earliest approaches that have been pursued to increase permeability of the BBTB. In particular, bradykinin and adenosine agonists have been extensively studied for this purpose and some of these compounds have progressed into clinical trials. This is the case of the bradykinin B2 receptor agonist cereport (RMP-7, lobradimil) [64], which has been used to transiently open the BBTB to different chemotherapeutics. After initial encouraging results [65,66], a phase II, randomized, controlled trial has shown that the addition of cereport (300 ng/kg) had no effect on the pharmacokinetics, toxicity or efficacy of carboplatin in patients with recurrent glioblastoma [67]. Subsequent pharmacokinetic studies suggested that higher doses of cereport may be required to increase carboplatin delivery to the tumor. However, a single-arm phase II trial failed to demonstrate any antitumor activity of the combination of 600 ng/kg of cereport and carboplatin in 38 children with varied brain tumors [68], and no further clinical trials with cereport in brain tumors have been reported.

Regadenoson (CVT-3146, Lexiscan) is an adenosine 2A receptor agonist that increased the BBB permeability in murine models in a dose-dependent and time-limited manner [69]. Clinical studies have failed to demonstrate the capacity of regadenoson (400 μg) to increase permeability of the integral BBB [70]. Likewise, a study aimed at enhancing regadenoson-induced increase of intratumoral temozolomide concentrations in glioblastoma patients yielded negative results [71].

The most likely explanation for the negative results obtained with these compounds in the clinics is their lack of specificity for the BBTB endothelium and their short biological half-life (e.g., 2–3 min for regadenoson). Increasing the dose administered may eventually yield the desired effect but at the possible expense of an undesirable, generalized increase of endothelial permeability. Despite these concerns, a clinical dose-finding study (from 50 to 1400 μg) has been scheduled to find the optimal regadenoson dose to increase BBB permeability (NCT03971734).

Tumor necrosis factor-alpha (TNF) is an inflammatory cytokine that has the potential to enhance the BBTB permeability. Indeed, preclinical studies on murine models of brain metastases have shown that systemic administration of TNF can permeabilize the BBTB to the anti-human epidermal growth factor receptor (HER) 2 mAb trastuzumab at tumor sites [72]. However, systemic administration of TNF is precluded because of its prohibitive systemic toxicity [73]. This may be bypassed by fusing TNF through its N-terminus to CNGRCG, a tumor vasculature-homing peptide that recognizes an isoform of membrane-bound aminopeptidase N (CD13) that is upregulated in the neovasculature of many solid tumors [74,75], including the vasculature of glioblastoma and primary CNS lymphoma [75,76,77]. The tumor vasculature-homing properties of the CNGRCG-TNF fusion protein (called NGR-TNF) allows the targeted delivery of extremely low, yet pharmacologically active doses of TNF to tumor blood vessels, thereby avoiding systemic toxicity (Figure 2B). Studies in various murine models of solid tumors have shown that picogram doses of NGR-TNF are sufficient to target the tumor vasculature, increase barrier permeability and, consequently, enhance drug penetration and efficacy [75,78,79]. On these bases, low-dose NGR-TNF has been used, alone or in combination with various chemotherapeutic agents, in patients with different types of solid tumors, with evidence of therapeutic efficacy and an excellent safety profile [73,80]. Relevant to the present context are the results of a phase II trial on 28 patients with relapsed or refractory primary CNS lymphoma, showing that NGR-TNF could alter the BBTB in a transient and reversible manner and increase the efficacy of R-CHOP (rituximab, cyclophosphamide, doxorubicin, vincristine and prednisone), a chemotherapy regimen consisting of drugs that very poorly cross the BBTB [76,77]. NGR-TNF-induced alteration of the BBTB was no longer detectable before the subsequent R-CHOP cycle three weeks later, thereby demonstrating the reversibility of its effects on the BBTB permeability. The effect of NGR-TNF on tumor vasculature permeability, which has been demonstrated by dynamic contrast-enhanced (DCE)-MRI and single-photon emission CT (SPECT) with diethylenetriaminepentaacetic acid tagged with ^99m^Tc (^99m^Tc-DTPA-SPECT), has been more evident in lymphomatous and peritumoral areas [76]. Moreover, CD13, the target of the NGR domain of this conjugate, has been found to be expressed by endothelial cells and pericytes of the brain tumor vasculature [76,77]. NGR-TNF has not impaired R-CHOP tolerability and no patient has interrupted treatment or needed dose reduction because of toxicity. The primary endpoint of the trial has been achieved, with an ORR of 75% and a median disease-free survival of 11 months (range 6–25+) [77]. These encouraging results contrast with limited benefit achieved with regadenoson and cereport, which may be explained, at least in part, by the widespread distribution of receptors to the latter drugs throughout the endothelial lining of the organism, whereas CD13 expression is more restricted to angiogenic/inflamed endothelia, including tumor blood vessels. While the use of this targeted version of TNF has yielded very positive results, a note of caution regarding the use of TNF in the CNS should be sounded in consideration of some recent results showing the negative effects of TNF on neurogenesis [81]. It is doubtful, however, that this may represent a major concern for NGR-TNF, which is delivered at very low doses to the brain tumor vasculature rather than to the neuronal components of the CNS. Furthermore, after a median follow-up of 2-years, no signs of neurotoxicity were observed in patients treated with NGR-TNF [77].

### 3.3. Approaches to Overcome the BBTB Exploiting Intrinsic Transport Mechanisms

**Figure 3:**
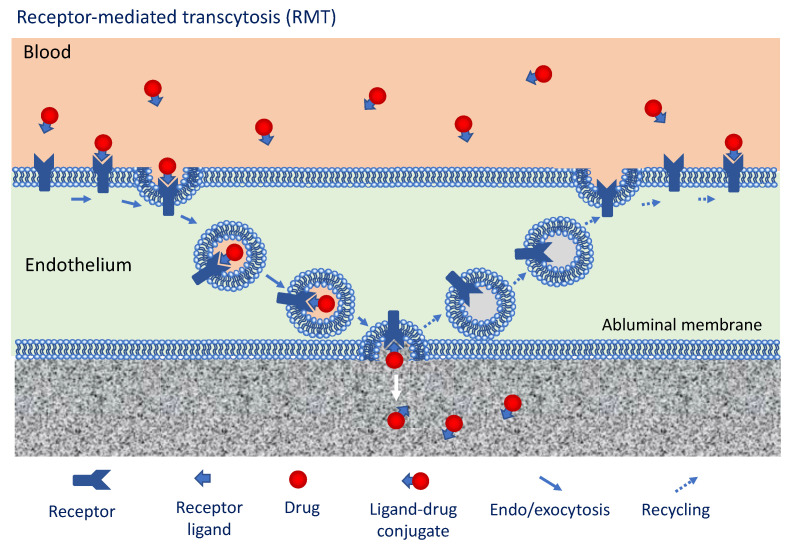
Receptor-mediated transcytosis (RMT) as a means to enhance drug delivery to brain tumors. This figure shows how receptors that are expressed on endothelial cells of the BBTB can be exploited to enhance drug delivery to the brain tumor. A selected ligand (peptide or antibody) against a given receptor (a recycling receptors as, e.g., the transferin receptor-TfR) is conjugated to a drug. The ligand-drug conjugated in the blood-stream is internalized through endocytosis and externalized into the brain tumor tissue through exocytosis. The receptor is then recycled to the luminal surface of the endothelial cell.

Enhancing drug delivery to the brain taking advantage of normal transport mechanisms through the BBTB is another approach that has been pursued. For this purpose, drugs are conjugated to molecules that use these transport mechanisms, without altering, even temporarily, the BBTB. Several routes have been investigated for this purpose, in particular adsorptive-mediated transcytosis (AMT), solute carrier-mediated transcytosis (SCMT) and receptor-mediated transcytosis (RMT). AMT is triggered by an electrostatic interaction between a cationic ligand and anionic proteoglycans expressed on the surface of the BBTB endothelium [7]. Cationic proteins [82] or cell-penetrating peptides [83,84] can be delivered with this approach. However, the main drawback of this approach is related to its inherent nonspecificity, owing to the fact target molecules are present also in normal vascular endothelia, and not exclusively in the BBTB. SCMT exploits the function of solute carriers as bidirectional transporters that carry their substrates down their concentration gradient from the luminal side to the abluminal side of the endothelial lining and vice versa [85]. Glucose transporters, large neutral amino acid transporters, organic anion-transporting polypeptides are examples of solute carriers that have been investigated to facilitate BBTB crossing by drugs [86,87,88]. AMT and SCMT are far away from being used in clinical neuro-oncology. Conversely, RMT is certainly the most deeply investigated and, theoretically, the most sophisticated route for shuttling drugs or candidate drugs into the brain. In RMT, a ligand is transported across the BBTB upon interaction with its specific receptor (Figure 3). This transport mechanism is not restricted by the size of the ligand and is suitable also for large molecules such as mAbs [89]. However, its transport capacity may be constrained by the number of receptors expressed per endothelial cell and this may not be balanced by the capacity of these receptors to recycle. The initial step occurring during RMT is a ligand-receptor interaction on the luminal side of the BBTB (Figure 3). This is followed by endocytosis of the ligand–receptor complex, endosome-mediated transport to the abluminal side of the BBTB and eventual exocytosis of the ligand [89]. Examples of receptors of this category are the insulin receptor [90], the transferrin receptor (TfR) [91], the low-density lipoprotein receptor (LDLR) [92], the diphtheria toxin receptor [93] and the nicotinic acetylcholine receptor [94]. The TfR has been intensively investigated because it is not only expressed on the endothelial cells of the BBTB but is also overexpressed on tumor cells, whether primary or metastatic tumors [95].

So far, results from clinical studies using the RMT route had disappointed the expectations, but, in recent times, two drug conjugates have progressed into clinical trials. The first uses Angiopep-2 as the RMT ligand. This is a 19-amino acid peptide that traverses the capillary endothelium of the BBTB upon interaction with the LDLR-related protein 1 (LRP1) [96]. LRP1 is expressed in a wide range of tissues and in multiple tumors [97]. Conditions of cellular stress, such as hypoxia and acidosis, upregulate its expression [97]. Functionally, LRP1 acts as a multifunctional scavenger receptor and contributes to the malignant phenotype of tumor cells [98]. ANG1005 (also termed GRN1005) comprises Angiopep-2 conjugated via cleavable ester bonds to three molecules of paclitaxel [99]. Systemically administered ANG1005 penetrated the brain parenchyma more rapidly and to a greater extent than unconjugated paclitaxel [99] and localized to orthotopic gliomas in mice expressing high levels of LRP1 [97]. In a phase I study, 36 patients with recurrent malignant glioma have received ANG1005 every 3 weeks [100]. Therapy was well tolerated with mucositis and neutropenia as DLTs. One CR, two partial responses (PR) and 8 stable diseases (SD), which lasted a median of 51 days, were recorded. Very recently, results of an open-label phase II study in adult patients with brain metastases from both HER2-positive or HER2-negative breast cancer, with or without leptomeningeal carcinomatosis, have been reported [101]. ANG1005 was administered intravenously at 600 mg/m^2^ every 3 weeks. Patient benefit (SD or better) was seen in intracranial and extracranial disease in, respectively, 77% and 86% of the evaluated patients, with an intracranial ORR of 15%. This result has not met the preset rule, but, when compared to historical control, symptom and OS improvement has been seen in patients with leptomeningeal carcinomatosis. ANG1005 is now investigated in phase III trials in a similar patient population with brain metastases from HER2-negative breast cancer (Table 3). Three other Angiopep-2-based conjugates for cancer therapy have been generated: ANG1007, carrying doxorubicin; ANG1009, carrying etoposide [102]; and ANG4043, carrying an anti-HER2 mAb [103]. ANG1007 and ANG4043 are now in preclinical development (company website inspected as of 2021/03/25).

A TfR-targeted construct designed to cross the BBTB is SGT-53, a cationic liposome formulation encapsulating a plasmid with the human tumor suppressor gene *TP53*. TfR is targeted by a single chain mAb fragment [104]. This nanoparticulate was shown to cross the BBTB and target intracranial glioblastoma xenografts. The combination of SGT-53 and temozolomide has limited development of resistance to this alkylating drug and had a prolonged antitumor effect in a model of temozolomide-resistant glioblastoma [105,106]. Results of a phase II study (NCT02340156) addressing the combination of SGT-53 and temozolomide in patients with recurrent glioblastoma are pending (Table 3). Interestingly, SGT-53 seems to boost antitumor immunity and sensitize glioblastoma to anti-PD-1 (anti-programmed cell death protein 1) therapy by upregulating PD-L1 expression [107], while reducing immune-related adverse events [108].

As a promising approach, a platform technology referred to as a BBB transport vehicle, consisting of a human IgG1 Fc engineered to bind to RMT targets expressed on the surface of brain endothelial cells, was recently developed [109,110]. TfR has been used as a BBB receptor target for proof-of-concept work to successfully deliver iduronate 2-sulfatase, the lysosomal enzyme deficient in mucopolysaccharidosis type II, to the brain in a preclinical model. The use of this ligand could likely be extended to primary or secondary brain tumors upon conjugation to antitumor drugs.

## 4. Conclusions

Overall, the last few years have represented a turning point in the development of efficacious approaches for traversing the BBTB and facilitating drug delivery to brain tumors. It seems likely that, in the near future, the more invasive approaches (e.g., CED, SACI, etc.) will be replaced by more patient-friendly and efficacious means. Enhancers of BBTB permeability, such as the US-based approaches or NGR-TNF, or refined approaches exploiting RMT for drug delivery have yielded very promising results in preclinical studies and, in some cases, also in early-stage clinical studies in patients with brain tumors. It is tempting to speculate that the combination of some of these approaches may give rise to even better additive or synergistic therapeutic effects.

## Figures and Tables

**Table 1:** Clinical trials with drugs directly delivered into the brain ^.

Drug	Clinical Indication	Phase	Clinicaltrials.Gov Number
*Placing drug during surgery*			
Surgery + Gliadel Wafer (carmustine) vs. Surgery + radiation therapy after surgery.	Metastatic brain disease	II	NCT04222062
Surgery with 5-ALA given together with Gliadel Wafer, followed by radiation therapy and temozolomide.	Glioblastoma	II	NCT01310868
Surgery + Gliadel Wafer	Metastatic brain cancer	II	NCT00525590
*Convection-enhanced delivery*			
Nanoparticle formulation of panobinostat (MTX110)	HGG (pontine)	I/II	NCT03566199 NCT04264143
Topotecan	HGG	I	NCT03154996 NCT03927274 NCT02278510
Carboplatin	HGG	I	NCT01644955
Liposomal formulation of irinotecan	HGG	I	NCT03086616 NCT02022644
Liposomal formulation of rhenium (186RNL)	Glioma	I/II	NCT01906385
^124^I-labeled anti-B7-H3 mAb 8H9	HGG (pontine) treated with radiation therapy	I	NCT01502917
D2C7 immunotoxin (scFv from the anti-EGFR mAb D2C7 linked to the *Pseudomonas* exotoxin PE38KDEL)	HGG	I	NCT02303678
D2C7-immunotoxin in combination with anti-PD-L1 mAb atezolizumab	HGG (recurrent)	I	NCT04160494
Anti-CD40 mAb (2141-V11) with D2C7-immunotoxin	Grade III/IV malignant glioma	I	NCT04547777
IL4 linked to a modified version of *Pseudomonas* exotoxin A (MDNA55)	HGG (recurrent or progressive)	I	NCT02858895
Bone morphogenetic protein (BMP) 4	HGG (progressive and/or recurrent)	I	NCT02869243
Safety study of replication-competent Adenovirus (Delta-24-rgd)	Recurrent glioblastoma	I/IIC	NCT01582516
Oncolytic poliovirus therapy with PVSRIPO	HGG (recurrent)	I	NCT03043391 NCT01491893
Oncolytic poliovirus therapy with PVSRIPO with anti-PD-1 mAb pembrolizumab	Glioblastoma	I	NCT04479241
GRm13Z40-2, an allogeneic CD8+ cytolitic T-cell line expressing IL13-Zetakine with IL-2.	Glioma and other brain tumors	IC	NCT01082926

^ The table reports clinical trials that are ongoing (updated not later than 5 years ago) or completed but without results being published in their entirety. Abbreviations: ALA, aminolevulinic acid; BMP, bone morphogenetic protein (BMP); HGG, high-grade glioma; IL, interleukin; mAb, monoclonal antibody; PD, programmed cell death; PVSRIPO, polio-rhinovirus chimera; scFv, single-chain variable fragment.

**Table 2:** Clinical trials based on increasing the permeability of the BBTB ^.

Osmotic Disruption/SIACI			
Drug(s)	Clinical Indication	Phase	Clinicaltrials.gov number
Cetuximab and Bevacizumab	Relapsed/refractory glioma in patients under 22.	I/II	NCT01884740
Repeated infusion of bevacizumab	Newly diagnosed glioblastoma Relapsed glioblastoma and anaplastic astrocytoma	I/II I/II	NCT01811498 NCT01269853
Repeated infusion of cetuximab	Newly diagnosed glioblastoma	I/II	NCT02861898
Temozolomide	Newly diagnosed and anaplastic astrocytoma	I	NCT01180816
Cetuximab	Relapsed glioblastoma and anaplastic astrocytoma	I	NCT01238237
Bevacizumab	Relapsed/refractory glioblastoma and anaplastic astrocytoma Recurrent glioblastoma	I I	NCT00968240 NCT02285959
**FUS**			
US-emitting device	Drug(s) and clinical indication	Phase	Clinicaltrials.gov number
ExAblate 4000 Type 2	With carboplatin in recurrent glioblastoma.	I/II I/II	NCT04440358 NCT04417088
	Safety and feasibility in opening the BBTB in malignant gliomas before or during standard of care therapy or surgery	NA NA NA NA NA NA	NCT03322813 NCT03551249 NCT03712293 NCT01473485 NCT03616860 NCT00147056
	Safety and feasibility in opening BBTB in brain tumors other than glioblastoma (e.g., metastases)	NA NA NA	NCT03714243 NCT03714243 NCT02343991 *
SonoCloud	Safety of opening BBTB in patients with recurrent glioblastoma before systemic carboplatin chemotherapy.	I/II	NCT02253212
	BBTB opening and administration of albumin-bound paclitaxel in recurrent GBM.	I/II	NCT04528680
	DLT of escalating numbers of ultrasound beams (Phase 1); safety and efficacy (Phase 2a expansion) in HGG	I/II	NCT03744026
	Safety and efficacy of BBTB opening with nivolumab ± ipilimumab in brain melanoma metastases	I/II	NCT04021420
NaviFUS system	Efficacy and safety with bevacizumab in recurrent glioblastoma	NA	NCT04446416
	Safety and feasibility of transient opening of the BBTB in recurrent glioblastoma	NA	NCT03626896

^ The table reports clinical trials that are ongoing (updated not later than 5 years ago) or completed but without results being published in their entirety. * Tumor type not specified. Abbreviations: BBTB, blood–brain tumor barrier; DLT, dose-limiting toxicity; FUS, focused ultrasound; GBM, glioblastoma; HGG, high-grade glioma; NA, not applicable; SIACI, superselective intraarterial cerebral infusion; US, ultrasound.

**Table 3:** Clinical trials based on approaches that exploit physiological transport mechanisms (RMT) ^.

Carrier–Drug Combination	Clinical Indication	Phase	Clinicaltrials.Gov Number
ANG1005 (Angiopep-2 conjugated to paclitaxel)	HER2^-^ breast cancer patients with newly diagnosed leptomeningeal disease and previously treated brain metastases	III	NCT03613181
	Breast cancer patients with recurrent brain metastases.	II	NCT02048059
	Patients with recurrent high-grade glioma with or without bevacizumab.	II	NCT01967810
	Breast cancer patients with recurrent brain metastases with or without trastuzumab.	II	NCT01480583
SGT-53 (cationic liposomes encapsulating plasmid for human tumor suppressor gene *TP53*)	In combination with irradiation and/or chemotherapy in pediatric patients with recurrent or progressive CNS malignancies	I	NCT03554707

Abbreviations: BBB, blood–brain barrier; CNS, central nervous system; HER2, human epidermal growth factor 2; RMT, receptor-mediated transcytosis. ^ The table reports clinical trials that are ongoing (updated not later than 5 years ago) or completed but without results being published in their entirety.

## Data Availability

Published studies were identified through a search of PubMed using the reported keywords alone or combined. No filters have been added regarding publication dates, article types (i.e., review, meta-analysis, clinical trial) and text availability (abstract or full text). However, the reviewed literature was limited to studies published in the English language in peer-reviewed, international journals.
